# The Effects of Aqueous Extract of Vaccinium Arctostaphylos Leaves on Blood Pressure in Renal Hypertensive Rats

**Published:** 2011-02-01

**Authors:** A Khalili, M B Khosravi, A A Nekooeian

**Affiliations:** 1 Department of Pharmacology, Shiraz University of Medical Sciences, Shiraz, Iran; 2 Cardiovascular Pharmacology Research Center, Shiraz University of Medical Sciences, Shiraz, Iran; 3 Department of Anesthesiology, Medical School, Shiraz University of Medical Sciences, Shiraz, Iran

**Keywords:** Vaccinium arctostaphylos, Rat, Blood pressure, Hypertension

## Abstract

**Background:**

The leaves of Vaccinium arctostaphylos (Qare qat) is advocated for the treatment of hypertension in Iran' folk medicine. The objective of was to examine the possible hypotensive activity of aqueous extract of Vaccinium arctostaphylos leaves in rat model of two-kidney, one-clip hypertension.

**Methods:**

Rats were subjected to sham operation of the placement of Plexiglass clip on left renal arteries. Four weeks later, renal artery clipped rats were given intravenous injection of normal saline or the extract at 10, 25, or 75 mg/kg, and mean blood pressure and heart rate were measured before and 20, 40 and 60 minutes after vehicle or drug administration.

**Results:**

Compared to sham group, renal artery clipped groups had a significantly higher mean blood pressure, heart and right kidney weights, lower left kidney weight and significantly indifferent heart rate. Compared to vehicle treatment, the extract at 75 mg/kg, but not at 10 or 25 mg/kg, did reduce the mean blood pressure at 20, 40 and 60 minutes after administration without changing the heart rate.

**Conclusion:**

The findings showed that at a higher dose the extract did have hypotensive activity without changing the heart rate. The exact hypotensive mechanism remains to be investigated.

## Introduction

The Vaccinium genus, from Ericacea family, includes nearly 450 species such as Vaccinium myrtillus (Bilberry), Vaccinium macrocarpon (Cranberry), Vaccinium angustifolium (Lowbush blueberry), Vaccinium erythrocarpum (Bearberry), Vaccinium ashei (Rabbiteye Blueberry), Vaccinium pallidum (Blue ridge blueberry), and Vaccinium arctostaphylos (Whortleberry). Such species have been reported to have beneficial pharmacological effects including reducing blood pressure,[1][2][3] infarct size following ischemia and reperfusion[3][4] apoptosis,[3] serum glucose[5][6][7] and serum lipids[8][9] as well as antioxidant[10][11] and urinary antiseptic activities.[10][12]Vaccinium arctostaphylos (Qare qat in Persian) is the only species that grows in Iran.[13] The berries and leaves of Vaccinium arctostaphylos have been reported to have serum glucose and lipid lowering activities.[14] In Iran's folk medicine, the berries of the plant are used for the treatment of diabetes and hypertension.[13][14][15] A recent analysis of the constituents of Vaccinium arctostaphylos ripped berries indicated that it had 3 major anthocyanins including delphinidin 3-O-b-glucoside, petunidin 3-O-b-glucoside and malvidin 3-O-b-glucoside.[13][16][17] Consistent with Iran's folk believe, delphinidin 3-O-b-glucoside was shown to have vasorelaxing activities.[18] However, no such an analysis has been performed on the constituents of the plant leaves.To the authors' knowledge, the traditional belief that Vaccinium arctostaphylos leaves might reduce the blood pressure has not been examined using up-to-date experimental techniques. Therefore, the present study aims at examining such a belief using rat model of two-kidney, one-clip hypertension.

## Materials and Methods

Twenty five grams of dried leaves of Vaccinium arctostaphylos, obtained from Gol-Chay company (Tehran, Iran), were ground to powder, and soaked in 50 ml distilled water for 48 hours in room temperature. Afterwards, the product was filtered, concentrated in a rotary, and dried in a vacuum desicator. The yield was about 25-30%.Male Sprague-Dawley rats (200-250 g), obtained from Animal Breeding Center of Shiraz University of Medical Sciences were subjected to sham operation (n=6) or placement of a plexiglass clip around left renal arteries (n=23) as described previously.[15] Briefly, under ketamine (60 mg/kg) and xylazine (8 mg/kg) anesthesia, a left flank incision was made, and left renal artery was exposed. The arteries were then dissected free of surrounding connective tissues, and solid plexiglass clips were placed around them. Antibiotic (penicillin G) powder (Jaber Ebne Hayyan, Tehran, Iran) was applied to the site of incision, and the abdominal wall and the skin were sutured using catgut and silk suture materials, respectively. Sham-operated rats were subjected to a similar procedure, but no clips were placed around renal arteries. The animals were then recovered from anesthesia, and were kept for 4 weeks under standard condition (temperature; 22±2º C, relative humidity; 50% and 12 hours light/dark cycle) with food (standard rat pellet) and water ad libitum.Four weeks later, animal were anesthetised with an intraperitoneal injection of sodium thiopenthal (80 mg/kg). The animals were then tracheotomized to facilitate breathing during the experiment. The right carotid arteries were cannulated using PE-50 polyethylen catheters connected to a pressure transducer (ADInstrument, Australia) for the measurement of arterial pressure. Left jugular veins were cannulated for vehicle or extract administrations. The animals were allowed 30 minutes to recuperate from surgical stress. Afterwards, a baseline measurement of arterial blood pressure and heart rate was performed. Left renal artery clipped rats were then divided into 4 groups (n=5-6 each), and assigned to receive an intravenous injection of normal saline (0.2 ml) as vehicle, or aqueous extract of Vaccinium arctostaphylos leaves at 10, 25 or 75 mg/kg in identical volumes. The blood pressure and heart rate were measured 20, 40 and 60 minutes afterwards. At the end of experiments, the animals were sacrificed and weights of kidneys and hearts were determined.Mean arterial blood pressure was calculated as diastolic pressure plus one third of plus pressure. The weights of heart and left and right kidneys were normalized to body weight. Data are presented as mean±SEM. Mean arterial blood pressure and heart rate were analyzed using Kruskall Wallis test followed by Dunn's test for pair wise comparisons. The body, heart or kidney weights were compared using one way analysis of variance (ANOVA) followed by Duncan's Multiple Range test for pair wise comparisons. A p value of ≤ 0.05 was considered statistically significant. Data analysis was performed using Sigmastat statistical software.

## Results

**Fig. 1:**
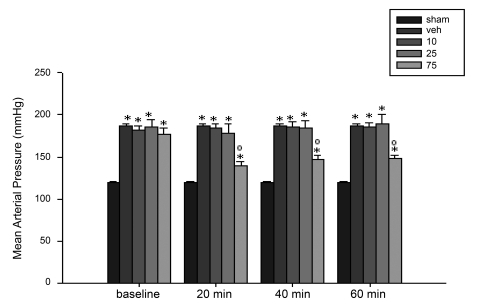
Blood pressure (mmHg) of sham-operated and renal artery clipped groups receiving vehicle (0.2 ml normal saline) or aqueous extract of arctostaphylos berries at 10, 25 or 75 mg/kg at the baseline and 20, 40 and 60 minutes after drug or vehicle administration.(*Denotes significant difference (P≤0.05) from sham-operated group; O Denotes significant difference (P≤0.05) from renal artery clipped rats receiving vehicle.)

**Fig. 2:**
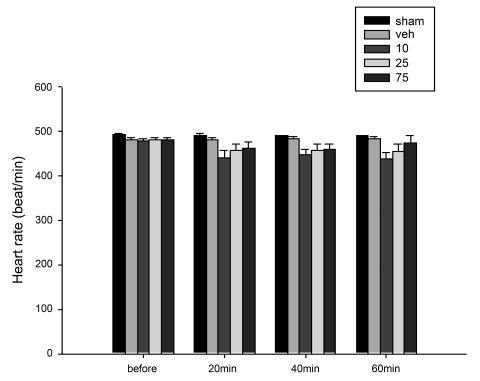
Heart rate (beats/min) of sham-operated and renal artery clipped groups receiving vehicle (0.2 ml normal saline) or aqueous extract of arctostaphylos berries at 10, 25 or 75 mg/kg at the baseline and 20, 40 and 60 minutes after drug or vehicle administration.

Four weeks after the operation, left renal artery clipped rats had a significantly higher mean arterial pressure than that of sham-operated rats (Figure 1). However, there was no significant difference between heart rate from sham-operated and left renal artery clipped rats (Figure 2). Moreover, the heart and right kidney weights of renal artery clipped rats were significantly higher than those of sham-operated rats, respectively. However, the weight of left kidney from renal artery clipped rats was significantly lower than that of sham-operated ones (Table 1).

**Table 1:** The weights (% of body weight) of left kidneys (LKW), right kidneys (RKW), hearts (HW) and body (BW) of sham-operated rats (Sham) and renal artery-clipped (RAC) rats receiving vehicle (RAC-Veh; normal saline; 0.2 ml), or aqueous extract of Vaccinium arctostaphylos berries at 10 (RAC-AE10), 25 (RAC-AE25) or 75 (RAC-AE75) mg/kg body weight.

** Sham**		**RAC-Vehicle**	**RAC-AE 10 mg/kg**	**RAC-AE 25 mg/kg**	**RAC-AE 75 mg/kg**
RK	0.40±.01	0.49±0.01^a^	0.47±0.01^a^	0.48±0.02^a^	0.49±0.01^a^
LK	0.38±0.01	0.19±0.02^a^	0.25±0.01^a^	0.20±0.02^a^	0.22±0.01^a^
HW	0.33±0.01	0.44±0.02^a^	0.43±0.01^a^	0.420±0.01^a^	0.43±0.02^a^
BW	240±7.3	249±9.3	244±0.6	240±5.7	245±15.6

^a^ Denotes significant difference from sham-operated group

There was no significant difference between baseline mean blood pressure of renal artery clipped group receiving vehicle, or the extract at 10, 25 or 75 mg/kg. Moreover, there was no significant difference between mean blood pressure of groups receiving the extract at 10 or 25 mg/kg and that of group receiving vehicle at 20, 40 or 60 minutes after administration. However, the mean blood pressure of group receiving the extract at 75 mg/kg was significantly lower than that of vehicle-treated group at 20, 40 and 60 minutes after administration.There was no significant difference between the heart rates of sham-operated groups or renal artery clipped groups receiving vehicle or the extract at 10, 25 or 75 mg/kg at 20, 40 or 60 minutes after administration (Figure 2).

## Discussion

The present study showed that placement of solid plexiglass clips on left renal artery resulted in hypertension characterized by increased mean arterial pressure as well as the weights of the heart and right kidney, and reduced left kidneys weight. It also showed that aqueous extract of Vaccinium arctostaphylos at 75, but not 10 or 25 mg/kg, did reduce mean arterial pressure without changing the heart rate.Two-kidney, one-clip model of experimental hypertension is one the widely-used models for the study of antihypertensive effects of various drugs or medicinal plants. This model has been induced in laboratory rats using silver clips.[20][21]

However, we have induced this model using solid plexiglass clips.[19][22].The present study also showed that aqueous extract of Vaccinium arctostaphylos leaves reduced blood pressure at the highest dose used. To the authors knowledge, this represents the first report on antihypertensive effects of Vaccinium arctostaphylos leaves extract. In agreement with the findings of his study, the berries of other species of Vaccinium were shown to have antihypertensive effects. Feeding of spontaneously hypertensive rats with diets containing 30% freeze-dried wild blueberry (Vaccinium angustifolium) for 8 weeks was associated with a reduction of blood pressure staring at 4 weeks of diet consumption.[1] Moreover, diet containing 3% of blueberries (Vaccinium ashei reade) leaves resulted in the reduction of blood pressure in spontaneously hypertensive rats.[23] In addition, the consumption of whole billberries (Vaccinium myrtillus) and crushed lingoberries for 8 weeks in human subjects was associated with a significant reduction in blood pressure.[24] Vaccinium macrocarpon (American cranberries) was also reported to have antihypertensive activities in human.[11]

The findings of this study can not speculate on the likely mechanisms underlying the hypotensive activity of the extract. However, the hypotensive activity of aqueous extract of blueberry (Vaccinium ashei reade) leaves[23] was attributed to angiotensin converting enzyme inhibition. In addition, other Vaccinium berries were reported to act via α1-antagonism[24] and angiotensin converting enzyme inhibition,[2][5][23] and endothelial nitric oxide release enhancement.[25][26] Whether or not, similar mechanisms do play a role needs to be investigated.The hypotensive activity of Vaccinium arctostaphylos extract in the present study was not accompanied by tachycardia reflex. Vasodilating agents including α1-antagonists and direct vasodilators,[27] are believed to induce reflex tachycardia, whereas angiotensin converting enzyme inhibitors do not cause such an effect,[27] Judging the findings of the present study along with that of others', that the leaves of other Vaccinium genus had angiotensin converting enzyme inhibition activities[23] might be suggestive of the involvement of a similar mechanism.From the findings of the present study, it is not possible to speculate as to what constituent of the plant leaves might be responsible for its antihypertensive effects and what the likely mechanisms would be. To the best of our knowledge, the chemical constituents of the Vaccinium leaves have not been investigated. However, previous publications on the Vaccinium genus might shed some light on the matter. Vaccinium arctostaphylos berries were reported to include anthocyanins namely, delphinidin 3-O-β-glucoside, petunidin 3-O-β-glucoside and malvidin 3-O- β-glucoside.[13][17] Moreover, Delphinidin 3-O-b-glucoside was shown to possess vasorelaxing activity.[19] The chemical constituents of the plant leaves and their possible involvements in the observed hypotensive activity are in need of further investigation.

In conclusion, the findings of the present study showed for the first time that aqueous extract of Vaccinium arctostaphylos leaves did have hypotensive activities without causing reflex tachycardia. The findings can not speculate on the likely mechanisms involved. The exact hypotensive mechanism of the extract needs to be investigated.
